# Common atrium and the associated malformations: Evaluation by low-dose dual-source computed tomography

**DOI:** 10.1097/MD.0000000000012983

**Published:** 2018-11-16

**Authors:** Yi Zhang, Zhi-gang Yang, Meng-xi Yang, Ke Shi, Rui Li, Kai-yue Diao, Ying-kun Guo

**Affiliations:** a Department of Radiology; b Department of Radiology, National Key Laboratory of Biotherapy, West China Hospital; c Department of Radiology, Key Laboratory of Birth Defects and Related Diseases of Women and Children of Ministry of Education, West China Second University Hospital, Sichuan University, Chengdu, China.

**Keywords:** computed tomography angiography, congenital, heart atria, heart defects, heart ventricles, sensitivity and specificity

## Abstract

Common atrium (CA) is a rare complex congenital heart disease. The studies of CA are mostly case reports, while few have been done regarding its morphological characteristics. We aimed to determine CA characteristics and diagnostic accuracy in assessing associated malformations in these patients with low-dose dual-source computed tomography (DSCT).

Twenty-one pediatric and adolescent CA patients underwent low-dose DSCT. Different ventricular types and associated malformations were assessed. The diagnostic accuracy of DSCT and transthoracic echocardiography (TTE) in evaluating associated malformations were assessed. The effective doses of DSCT were calculated.

Patients (n = 21) were divided into CA with biventricular physiology (n = 7) and CA with single ventricle (SV) (n = 14). There were 3 types of SV morphology: single left ventricle (n = 5), single right ventricle (n = 6), and undifferentiated ventricle (n = 3). In all, 22 associated malformations were seen in CA and 56 in CA with SV. DSCT was superior to TTE for detecting intracardiac anomalies (sensitivity: DSCT, 92.31% vs TTE, 76.92%), great vessels anomalies (sensitivity: DSCT, 100.00% vs TTE, 77.50%), and of collateral vessels (sensitivity: DSCT, 100% vs TTE, 20.00%). The estimated mean effective dose was 0.95 ± 0.44 mSv (<1 mSv).

This study indicated that low-dose DSCT is an ideal alternative for pediatric and adolescent patients with CA, providing morphological details of CA and associated malformations with high accuracy.

## 1 Introduction

Common atrium (CA) is defined as the condition of complete absence of the atrial septum. The mixture of arterial and venous blood in the CA usually causes palpitations, dyspnea on effort, and mild cyanosis.^[1]^ In CA patients with single ventricle (SV), interventricular communication may further aggravate the hemodynamic instability and cause severe cyanosis and hypoxia.^[2]^ It has been reported that CA patients with more malformations and more severe clinical features tend to receive surgery earlier than those with CA alone.^[1–4]^ However, most of the reports are cases. The morphological characteristics of CA and the differences of associated anomalies still need to be clarified. Furthermore, accurate diagnosing of CA and its accompanied malformations preoperatively contributes to making appropriate surgery plans.

Transthoracic echocardiography (TTE) has been used as the first-line imaging modality for patients with congenital heart disease. However, the poor acoustic window may limit visualization of cardiovascular structures, especially for extracardiac malformations.^[5,6]^ In recent years, dual-source computed tomography (DSCT) has frequently been applied in complex congenital heart disease for its high spatial resolution, low radiation dose, and powerful data post-processing techniques such as multiplanar reformation, maximum intensity projection, and volume rendering. Several studies have demonstrated the diagnostic accuracy of DSCT and cardiac magnetic resonance in complex congenital heart disease.^[7–10]^ To the best of our knowledge, however, the studies of CA are mostly case reports, while few have been done regarding its morphological characteristics and the diagnostic accuracy of DSCT in estimating CA.^[2–4,11–15]^ Therefore, we enrolled 21 pediatric and adolescent patients to determine characteristics of and diagnostic accuracy for CA in assessing associated multiple malformations in these patients with low-dose DSCT.

## 2 Methods

### 2.1 Study population

We retrospectively enrolled 39 pediatric and adolescent patients with CA admitted to our hospital between July 2008 and June 2018. The diagnosis of CA was confirmed by DSCT, TTE, and surgical results.^[1,16]^ Exclusion criteria were patients with unstable clinical conditions (n = 5) and without surgical intervention (n = 13). Finally, 21 patients (9 males and 12 females; average age 5.8 ± 5.0 years, range 0.5–16.3 years) underwent low-dose DSCT and TTE. This study was approved by the Institutional Review Board at our institution (No. 14–163), and patient consent was waived due to the retrospective nature of this study. All sensitive information of patients was treated confidentially and used only for the purpose of this study.

### 2.2 Image acquisition

Scans were performed on a DSCT scanner (Somatom Definition; Siemens Medical Solutions, Forchheim, Germany) 3 days before the surgery. Short-term sedative (chloral hydrate with a concentration of 10%, 0.5 mL/kg) was given to the patients under the age of 6 before the DSCT examinations. The older patients were asked to hold their breath while scanning. All patients underwent DSCT with a retrospectively ECG-gated protocol. The ECG-pulsing window was set to Auto. The acquisition parameters were as follows: 80 kV tube voltage, 100 mAs tube current, 0.28 s gantry rotation time, and a pitch of 0.2 to 0.5 (selected according to the heart rate; a higher pitch was used for higher heart rates). Scanning was performed in the craniocaudal direction, from the inlet of the thorax all the way to 2 cm below the diaphragm level. The nonionic contrast agent (iopamidol, 370 mg/mL; Bracco, Italy) was injected at a speed of 1.2 to 2.5 mL/s through an antecubital vein, followed by 20 mL of saline solution in the same way. The injected volume was adjusted based on the body weight (1.5 mL/kg). Bolus tracking was used in the region of interest (ROI) in the descending aorta with a predefined threshold of 100 HU. Image acquisition was triggered 5 seconds after the ROI attenuation reached the 100 HU threshold.

All ultrasonic examinations were performed on a Philips SONOS 7500 ultrasound system (Philips Medical Systems, Bothell, WA) right after the CT scan. In line with the recommendations of the American Society of Echocardiography Committee, the examination included M-mode, 2-dimensional, continuous wave, and color Doppler flow imaging.^[17]^ An experienced echocardiographer who was not involved in the diagnostics analyzed the TTE in a blind fashion.

### 2.3 Image analysis

All acquired data were transferred to a workstation (Syngo; Siemens Medical System, Forchheim, Germany). Images were reconstructed with a slice thickness of 0.75 mm and an increment of 0.7 mm. Two qualified radiologists (Y.Z. and M.X.Y.) analyzed the morphological characteristics of CA and the associated malformations preoperatively. According to the ventricular type, CA was further classified into 2 groups: CA and CA with SV. Any disagreement was referred to a third experienced radiologist to achieve consensus on the diagnosis.

**Figure 1:**
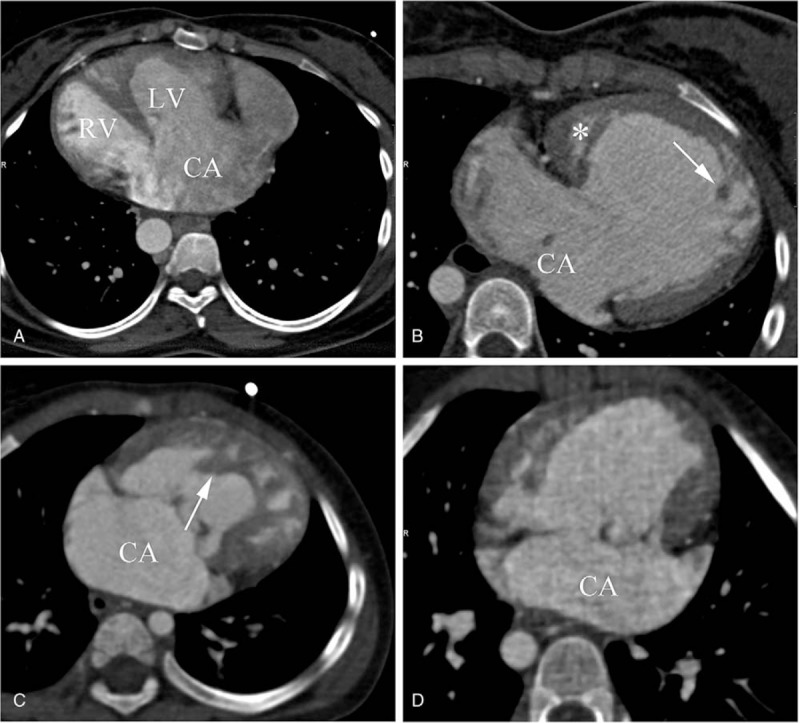
Different types of CA based on the morphological characteristics of ventricle. (A) CA with biventricular physiology. The CA is both connected to LV and RV. (B) CA with single LV. The trabeculae (arrow) of main ventricular chamber are thin and there is a rudimentary chamber (∗) located in the right side of the main chamber. (C) CA with single RV. The trabeculae (arrow) of main ventricular chamber are coarse. (D) CA with indeterminate ventricle. Single ventricle chamber is of indeterminate morphology. CA = common atrium; LV = left ventricle, RV = right ventricle, SV = single ventricle.

We then divided the ventricular morphology of SV into 3 types according to the basic pattern of SV morphology^[18]^: single left ventricle (single LV); single right ventricle (single RV), and indeterminate ventricle (Fig. 1).

In order to compare the diagnostic accuracies of DSCT and TTE regarding multiple associated malformations, we categorized all malformations by surgical result into the following 3 groups: intracardiac malformations, anomalies of great vessels, and of aortopulmonary collaterals. More specifically, intracardiac anomalies included cardiac malposition, atrial isomerism, complete atrioventricular septal defects, and single atrioventricular valve. A levoverted heart, dextrocardia, and mesocardia were considered as cardiac malpositions. Anomalies of great vessels included persistent left superior vena cava, anomalous pulmonary venous connection, right aortic arch, pulmonary artery anomalies, and patent ductus arteriosus.

### 2.4 Radiation dose estimation

Two radiation dose parameters, volume CT dose index (CTDI_vol_) and dose-length product (DLP), were obtained from the information generated automatically by the CT console after the examination. The effective dose (ED) was calculated from the DLP multiplied by age-specific DLP conversion coefficients. These conversion coefficients were 0.026 for patients between 4 months and 1 year of age, 0.018 for patients between 1 and 6 years of age, and 0.012 for patients > 6 years of age.^[19,20]^ In this study, patients older than 6 years were evaluated as 1 group.

### 2.5 Statistical analysis

Data were analyzed using SPSS software for Windows (version 19.0, SPSS Inc., Chicago, IL). Continuous variables were expressed as mean ± standard deviation. Categorical variables were expressed as numbers and percentages. The sensitivity, specificity, positive predictive value, and negative predictive value of DSCT and TTE were evaluated for the groups of intracardiac malformations, anomalies of great vessels, of collateral vessels, and of the coronary arteries.

## 3 Results

### 3.1 Population characteristics

**Figure 2:**
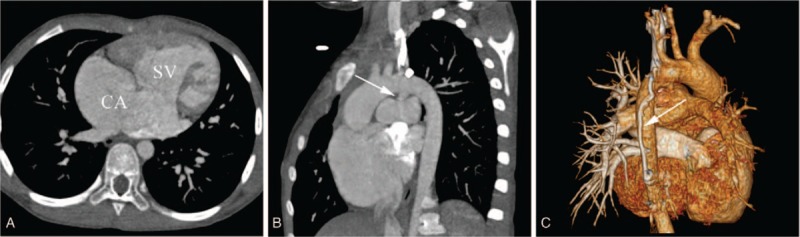
CA with single right ventricle in a female aged 9 years. (A) Axial image reveals a CA connected to one ventricular chamber with coarse trabeculae. (B) MPR image shows that a funnel-shaped patent ductus arteriosus arise from the aorta arch into the left pulmonary artery (arrow). (C) VR image showed a collateral vessel laying on the back of the descending aorta (arrow). CA = common atrium, MPR = multiple planar reconstruction, SV = single ventricle.

**Figure 3:**
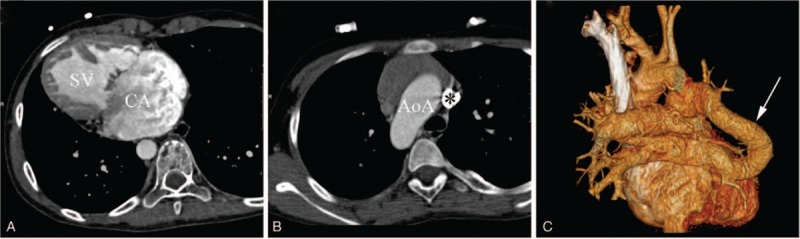
CA with indeterminate ventricle in a female aged 11 years. (A) Axial CT image reveals a CA with SV accompanied by dextrocardia. The morphological characteristics of SV cannot be identified. (B) Axial CT image shows a right-sided AoA and a persistent left superior vena cava (∗). (C) VR image shows a drainage of left pulmonary veins and partial right pulmonary veins into the right superior vena cava. AoA = aortic arch, CA = common atrium, SV = single ventricle, VR = volume reconstruction.

Twenty-one CA patients were finally included. Patients had a median age of 5.8 ± 5.0 years and there were 9 males and 12 females. Good-quality images with clear visualization of the anatomic details were obtained from all patients for imaging analysis. Seven CA patients with biventricular physiology (33.3%) and 14 CA with SV physiology (66.7%) (Figs. 2 and 3) were identified. Among the CA patients with SV, there were 3 types of SV morphology: single LV (n = 5), single RV (n = 6), and undifferentiated ventricle (n = 3) (Table 1). Of all the 78 associated malformations, 22 (28.2%) were seen in CA, while 56 (71.8%) were seen in CA with SV. The most common intracardiac malformation was single atrioventricular valve (n = 10, 12.8%). Two of the most common anomalies of great vessels were pulmonary artery anomalies (n = 15, 19.2%) and persistent left superior vena cava (n = 12, 15.4%) (Table 1).

**Table 1:**
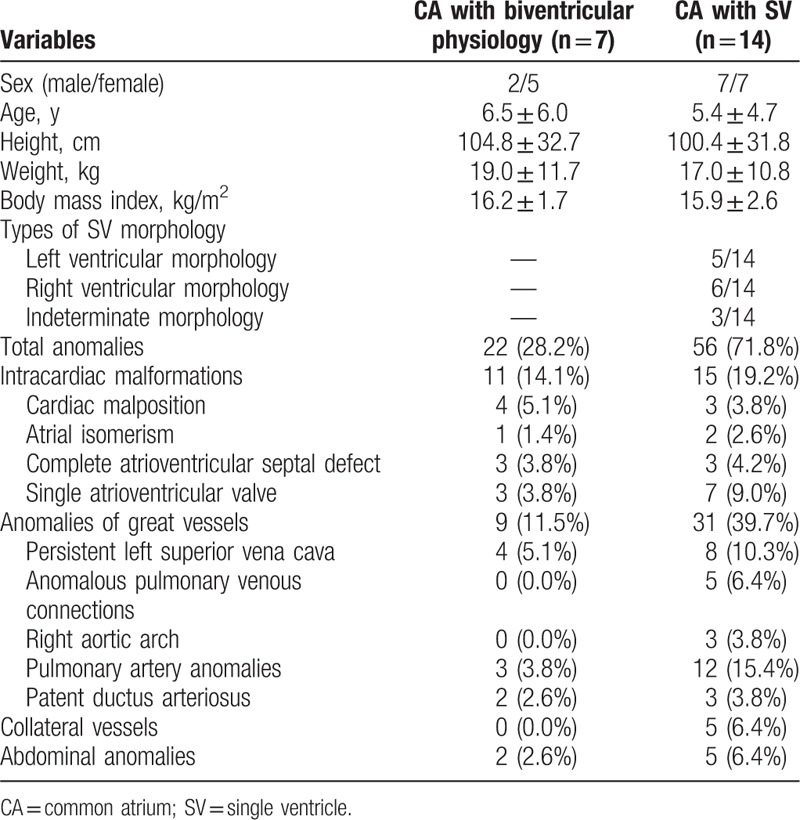
Baseline characteristics.

### 3.2 Diagnostic accuracy between DSCT and TTE

A total of 20 associated malformations were missed by either CT or TTE, including intracardiac malformations (DSCT: n = 1 vs TTE: n = 6), anomalies of great vessels (DSCT: n = 0 vs TTE: n = 9), and collateral vessels (DSCT: n = 0 vs TTE: n = 4) (Table 2). DSCT was superior to TTE for the detection of intracardiac anomalies (sensitivity: DSCT, 92.31% vs TTE, 76.92%; specificity: DSCT, 100.00% vs TTE, 75.00%), anomalies of great vessels (sensitivity: DSCT, 100.00% vs TTE, 77.50%; specificity: DSCT, 100.00% vs TTE, 87.69%), and collateral vessels (sensitivity: DSCT, 100% vs TTE, 20.00%; specificity: DSCT, 100.00% vs TTE, 100.00%) (Table 3). In addition, DSCT detected 7 patients with situs inversus during chest scans.

**Table 2:**
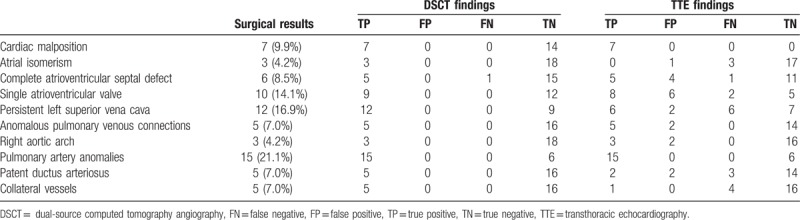
The summary of the findings obtained with DSCT and TTE.

**Table 3:**
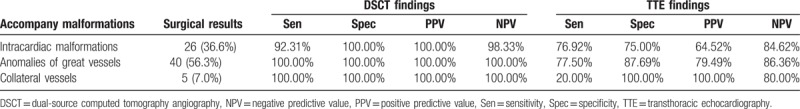
The diagnostic accuracies of DSCT and TTE according to anomalies categories.

### 3.3 Radiation dose

The mean DLP for patients between 4 months and 1 year of age was 43.25 ± 24.36 mGy, which corresponds to an estimated mean ED of 1.13 ± 0.63 mSv. The mean DLP for patients between 1 and 6 years of age was 52.00 ± 25.94 mGy, corresponding to an estimated mean ED of 0.94 ± 0.43 mSv. For patients older than 6 years, the values were 68.33 ± 12.06 mGy and 0.82 ± 0.15 mSv, respectively. The overall estimated mean ED was 0.95 ± 0.44 mSv (<1 mSv). The CTDI_vol_ was 5.17 ± 2.48 mGy for patients of 4 months to 1 year, 8.30 ± 4.20 mGy for 16 to six years, and 9.29 ± 4.26 mGy for older than 6 years.

## 4 Discussion

CA patients without multiple malformations usually have a relatively long life expectancy, even without surgical intervention.^[21,22]^ However, those with associated malformations who do not receive surgical treatment usually have a poor prognosis.^[2–4]^ Delayed identification of CA increases the possibility of pulmonary vascular disease, progression to arrhythmia, even cardiac dysfunction.^[21]^ Thus, the prompt diagnosis of CA, of the different ventricular types, and of the associated malformations contributes to the clinical treatments.

In CA patients, the atrial septum is completely absent. The dextral part of the CA has the morphology of a right atrium (crista terminalis, pectinate muscle, right atrial appendage) and receives the superior and inferior vena cava and coronary sinus. The sinistral portion of the CA has the morphology of a left atrium (smooth nontrabeculated walls, a left atrial appendage) and receives the pulmonary vein.^[16]^ In CA patients with SV, the CA connects to 1 ventricular chamber by either two separate atrioventricular valves or a common atrioventricular valve.^[18]^ More specifically, the SV morphology is divided into left, right, and indeterminate (Fig. 1). The single LV has relatively thinner trabeculae, while the single RV is more coarsely trabeculated.^[18]^ Our findings showed that CA with single RV is the most common morphology type, followed by CA with single LV and CA with undifferentiated ventricle is the least common type. In addition, more patients had CA with SV (14, 66.7%) than had only CA (7, 33.3%). This is consistent with previous reports in which patients with CA and SV admitted to hospital and received surgery at very young age.^[2–4]^ In part, this is because the clinical presentation of CA patients with SV is more complex, leading to relatively high detection and some CA patients without severe clinical manifestations may not be referred to hospital. Thus, more studies are still needed to determine the different prevalence of CA and CA with SV. In our study, malformations associated with CA with SV occurred in total approximately twice more than with CA, indicating that CA with SV tends to be associated with more malformations.

According to our study, DSCT was superior to TTE (sensitivity: DSCT, 92.31% vs TTE, 76.92%) or the detection of intracardiac anomalies. TTE missed more intracardiac malformations, for instance atrial isomerism, than DSCT. TTE also resulted in more misdiagnoses than DSCT, such as atrial isomerism, complete atrioventricular septal defects, and single atrioventricular valve. The reason is that regarding morphological evaluation, DSCT has an advantage over TTE due to its high spatial resolution, while TTE is better at the evaluation of cardiac function and valve movement.

In addition, our data indicated that DSCT was more suitable than TTE for detecting extracardiac malformations, including anomalies of great vessels and of collateral vessels. Furthermore, DSCT also detected seven separate thoracic and abdominal anomalies in chest scans. This is consistent with previous studies.^[7,8,11,12]^ The reasons for this are an extensive field of view of the thorax and abdomen given by DSCT and the addition of contrast. Contrarily, the diagnostic value of TTE, particularly for the distal segment of great vessels and separate abdominal anomalies, is limited by the limited range of its 3-dimensional reconstructions.^[23]^

As pediatric and adolescent patients are more radiosensitive than adults,^[24]^ several precautions were taken in our study to reduce radiation dose without decreasing the image quality. First, the tube voltage was set at a low level of 80 kV, which helps achieving higher vascular enhancement without loss of contrast-to-noise ratio.^[25]^ In addition, an ECG-based tube current modulation technique and heart rhythm adaptive pitch were applied to further reduce radiation exposure and shorten the duration of examinations.^[26,27]^ In this study, the mean ED was 1.13 ± 0.63 mSv for patients between 4 months and 1 year of age, 0.94 ± 0.43 mSv or patients between 1 and 6 years of age, and 0.82 ± 0.15 mSv for patients older than 6 years.

Our study had several limitations. First of all, our study is a single-center study and CA is an infrequent congenital heart disease. Thus, more studies in different populations are still required. Second, few follow-ups were conducted because the focus of this study was on the diagnostic accuracy of DSCT in overall evaluations of CA. The relationship between detection with DSCT and prognosis will be discussed in a future study. Finally, it is hard to avoid the selection bias, as this is a retrospective study.

## 5 Conclusion

CA is a rare condition that is reported as cases mostly. We collected 21 patients to investigate the morphological characteristics of CA. Low-dose DSCT is an ideal alternative for pediatric and adolescent patients with CA, providing morphological details of CA and associated malformations with high accuracy.

## Author contributions

Y.Z. and M.X.Y. should be considered cofirst authors. Z.G.Y. and Y.K.G. should be considered co-corresponding authors. Y.Z. participated in the study design, contributed to data analysis and interpretation, and drafted the manuscript. Z.G.Y. contributed to preparation, editing, and review of the manuscript. M.X.Y. carried out data acquisition, performed data analysis and interpretation, and edited the manuscript. K.S. and R.L. participated in data analysis and interpretation. K.Y.D. contributed to quantitative data analysis and preparation of the manuscript. Y.K.G. contributed to quality control of data and algorithms, and editing and review of the manuscript. All authors read and approved the final manuscript.

**Conceptualization:** Yi Zhang.

**Data curation:** Yi Zhang, Meng-xi Yang, Rui Li.

**Formal analysis:** Yi Zhang, Ke Shi, Kai-yue Diao.

**Funding acquisition:** Ying-kun Guo.

**Software:** Yi Zhang.

**Supervision:** Zhi-gang Yang.

**Writing – original draft:** Yi Zhang.

**Writing – review & editing:** Zhi-gang Yang, Kai-yue Diao.
